# Supplementary material for: Content-rich biological network constructed by mining PubMed abstracts
Source: BMC Bioinformatics. 2004 Oct 8;5:147. doi: 10.1186/1471-2105-5-147 (PMC528731; doi:10.1186/1471-2105-5-147)
Supplement: Additional File 5 — The original Chilibot query results of the term "long-term potentiation (LTP)" and 22 other terms, limiting the latest references analyzed to the years 1990, 1995, 2000, and 2004. [file 1471-2105-5-147-S5.bz2 › chilibotAdditionalFile5/ltp2000/html/ERK_CREB.html]

 


 **ERK** and **CREB** 
  
Found 42 abstracts in PubMed,  **30 abstracts were retrieved and analyzed**.  


---

 Search Google  |
 PDF files only 
|  EDU domain only 

---

**Interactive relationship** (e.g. stimulation, inhibition, etc)

- Activation of the extracellular regulated kinase  **ERK**  pathway occurs in stress responses and is involved in  **CREB**  activation.  Ref: 11123332 J Immunol, 2001
- In this report, we demonstrate that apoE4 stimulates the transcriptional activity of cAMP response element binding protein  **CREB**  by activating the extracellular signal regulated kinase  **ERK**  cascade in rat primary hippocampal neurons.  Ref: 11042199 J Biol Chem, 2001
- In contrast, cAMP phosphorylated  **CREB**  via PKA directly but required a Rap1  **ERK**  pathway to activate a component downstream of  **CREB**  phosphorylation and  **CREB**  binding protein recruitment.  Ref: 10950954 J Biol Chem, 2000
- ATF1 and  **CREB**  can be phosphorylated by Rsk2 which is a protein kinase directly activated by  **Erk**  MAP kinases.  Ref: 10723128 Oncogene, 2000
- We also show that inhibition of the MAPK  **ERK**  cascade by the MAPK kinase MEK inhibitor SL327 prevented  **CREB**  and Elk 1 phosphorylation, and LTP dependent gene induction, resulting in rapidly decaying LTP.  Ref: 10844026 J Neurosci, 2000
- Furthermore, using luciferase reporter gene assays, we found that the RAS  **ERK**  and PI3 K signaling pathways are important for activation of  **CREB**  and NF kappaB in GDNF treated cells, respectively.  Ref: 11002419 Oncogene, 2000
- Signaling proceeds through the p38 MAPK, the  **ERK**  MAPK, and the PKA signaling pathways, which leads to cumulative phosphorylation and activation of  **CREB** .  Ref: 10077003 Mol Endocrinol, 1999
- These results suggest a signaling pathway in which  **Erk**  MAP kinase activates the c fos enhancer by direct phosphorylation of p62TCF and by activation of Rsk related kinases that phosphorylate ATF1 and  **CREB** .  Ref: 10723128 Oncogene, 2000
- Multiple signaling pathways have been shown to contribute to the regulation of  **CREB**  dependent transcription, including both protein kinase A PKA and mitogen activated protein MAP kinase extracellular signal regulated kinase  **ERK**  dependent kinase cascades.  Ref: 10950954 J Biol Chem, 2000
- A common intermediate pathway initiating from cytokine receptors is the Ras Raf MEK  **ERK**  MAPK cascade, which can result in the phosphorylation and activation of additional downstream kinases and transcription factors such as p90Rsk,  **CREB** , Elk and Egr 1.  Ref: 10637471 Leukemia, 2000
- bFGF stimulation led to phosphorylation of the p38 mitogen activated protein kinase, and the extracellular signal regulated kinase MAPK and promoter activation, phosphorylation of  **CREB** , and GAL4  **CREB**  dependent transcription were selectively prevented by a dominant negative Ras mutant, the p38 MAPK specific inhibitor SB203580, and the MAP  **ERK**  kinase 1 inhibitor PD098059.  Ref: 10077003 Mol Endocrinol, 1999
- Moreover, these effects of ganoderma might be mediated via the ras extracellular signal regulated kinase  **Erk**  and cAMP response element binding protein  **CREB**  signaling pathways, as demonstrated by the phosphorylation of Erk1, Erk2 and  **CREB** .  Ref: 11119721 FEBS Lett, 2000
- Thus, activation of  **Erk**  may not be essential for neuronal differentiation in F11 cells and alphao may cause changes in NOG by inhibiting  **CREB**  activation.  Ref: 10617116 J Neurochem, 2000

**Parallel relationship** (e.g. studied together, co-existance, homology, etc.)

- Thus, MAPK  **ERK**  activation is required for LTP dependent transcriptional regulation and we suggest this is regulated by two parallel signaling pathways, the MAPK  **ERK**  Elk 1 pathway targeting SRE and the MAPK  **ERK**   **CREB**  pathway targeting CRE.  Ref: 10844026 J Neurosci, 2000
- In primary cultures of striatal neurons, glutamatergic activation of  **ERK**  and one of its transcription factor targets,  **CREB** , showed a calcium dependence typical of NMDA receptor mediated responses.  Ref: 10349832 J Neurochem, 1999
- Calcium mediated phosphorylation of  **CREB**  through the PKA Rap1  **ERK**  pathway.  Ref: 10950954 J Biol Chem, 2000
- ... the increase in  **CREB**  phosphorylation was not, which suggests that in area CA3 the PKA cascade is not coupled to  **CREB**  phosphorylation via  **ERK** .  Ref: 10777769 J Neurosci, 2000
- In contrast, apoE3 was unable to stimulate  **CREB**  transcriptional activity and unable to activate the  **ERK**  pathway.  Ref: 11042199 J Biol Chem, 2001
- Using the PC12 cell model system, we found that both calcium influx and cAMP stimulated  **CREB**  dependent transcription via a Rap1  **ERK**  pathway, but this regulation occurred through distinct mechanisms.  Ref: 10950954 J Biol Chem, 2000
- Furthermore, LPS plus CHX enhanced phosphorylation of  **ERK** , p38, and  **CREB**  in a synergistic manner.  Ref: 10924699 Brain Res, 2000
- Following a similar time course, the two downstream transcriptional targets of MAPK  **ERK** , cAMP response element binding protein  **CREB**  and the ternary complex factor Elk 1, a key transcriptional regulator of serum response element SRE driven gene expression, were hyperphosphorylated and the immediate early gene zif268 was upregulated.  Ref: 10844026 J Neurosci, 2000
- Genes regulated by  **CREB**  or other transcription factor targets of the CaMK, Ras  **ERK** , and SAPK pathways could mediate important adaptive responses to changes in synaptic activity such as changes in synaptic strength and the regulation of neuronal survival and death.  Ref: 10491574 J Neurosci Res, 1999
- Our results suggest that the phosphorylation of  **ERK** , p38, and  **CREB**  may be involved in the regulation of synergistic c fos mRNA expression induced by LPS plus CHX in C6 rat glioma cells.  Ref: 10924699 Brain Res, 2000
- Given the possible role of altered gene expression in the late phase of LTP and in long term memory, we evaluated the capacity of the MAPK  **ERK**  extracellular signal regulated kinase to regulate phosphorylation of the transcription factor cAMP response element binding protein  **CREB**  in hippocampal area CA1.  Ref: 11016081 Acta Neurobiol Exp (Wars), 2000
- Oxidative stress differentially modulates phosphorylation of  **ERK** , p38 and  **CREB**  induced by NGF or EGF in PC12 cells.  Ref: 10588574 Neurobiol AgingNeurobiol Aging, 2000
- However, incubation of slices with forskolin, an activator of the cAMP dependent protein kinase PKA cascade, did result in increases in active  **ERK**  and cAMP response element binding protein  **CREB**  phosphorylation in area CA3.  Ref: 10777769 J Neurosci, 2000
- To determine if  **CREB**  and mitogen activated protein kinases are involved in the regulation of c fos mRNA expression by LPS and CHX, Western blot was carried out using the phosphorylated form of antibodies against  **ERK** , JNK, p38, and  **CREB** .  Ref: 10924699 Brain Res, 2000
